# The association of mammographic density with ductal carcinoma *in situ *of the breast: the Multiethnic Cohort

**DOI:** 10.1186/bcr1507

**Published:** 2006-06-23

**Authors:** Jasmeet K Gill, Gertraud Maskarinec, Ian Pagano, Laurence N Kolonel

**Affiliations:** 1 Cancer Research Center of Hawaii, Honolulu, Hawaii, USA

## Abstract

**Introduction:**

It is well established that women with high mammographic density are at greater risk for breast cancer than are women with low breast density. However, little research has been done on mammographic density and ductal carcinoma *in situ *(DCIS) of the breast, which is thought to be a precursor lesion to some invasive breast cancers.

**Method:**

We conducted a nested case-control study within the Multiethnic Cohort, and compared the mammographic densities of 482 patients with invasive breast cancer and 119 with breast DCIS cases versus those of 667 cancer-free control subjects. A reader blinded to disease status performed computer-assisted density assessment. For women with more than one mammogram, mean density values were computed. Polytomous logistic regression models were used to compute adjusted odds ratios (ORs) and 95% confidence intervals (CIs) for two measurements of mammographic density: percentage density and dense area.

**Results:**

Mammographic density was associated with invasive breast cancer and breast DCIS. For the highest category of percentage breast density (≥50%) as compared with the lowest (<10%), the OR was 3.58 (95% CI 2.26–5.66) for invasive breast cancer and 2.86 (1.38–5.94) for breast DCIS. Similarly, for the highest category of dense area (≥45 cm^2^) as compared with the lowest (<15 cm^2^), the OR was 2.92 (95% CI 2.01–4.25) for invasive breast cancer and 2.59 (1.39–4.82) for breast DCIS. Trend tests were significant for invasive breast cancer (*P *for trend < 0.0001) and breast DCIS (*P *for trend < 0.001) for both percentage density and dense area.

**Conclusion:**

The similar strength of association for mammographic density with breast DCIS and invasive breast cancer supports the hypothesis that both diseases may have a common etiology.

## Introduction

As a result of increasing early detection efforts, breast carcinoma *in situ *(CIS) constitutes more than 20% of newly diagnosed breast cancer cases in the USA [1]. Although breast CIS shares a number of risk factors with invasive breast cancer, and ductal carcinoma *in situ *(DCIS) of the breast is considered to be a precursor to some invasive breast cancers [2], it is not clear to what extent breast CIS and invasive breast cancer have the same etiology. Several case-control studies have confirmed that mammographic density is associated with risk for breast cancer [3]; women with high breast density have a three-fold to six-fold greater risk for developing breast cancer than women with low breast density [3,4]. Most studies of mammographic density included only invasive breast cancer cases [5] or combined invasive and CIS cases [6-10]. One study investigating breast CIS observed that DCIS was more likely to occur in mammographically dense areas [11], and another study reported an increase in breast hyperplasia or atypia/CIS in women with greater than 50% breast density [12]. This analysis examines the association between mammographic density and risk for breast DCIS in comparison with invasive breast cancer and breast cancer-free control subjects.

## Materials and methods

### Study population

The data for this analysis were collected using a nested case-control study within the Hawaii component of the Multiethnic Cohort, which was established between 1993 and 1996 [13]. As described in detail elsewhere [4], all female members diagnosed with primary breast cancer between cohort entry and December 2000 were identified as potential cases (*n *= 1,587). A similar number of randomly selected control subjects (*n *= 1,584) who were not known to have breast cancer were frequency matched to the distribution of ethnicity and 5-year age groups of the cases. Cases and controls with a previous diagnosis of breast cancer, a history of breast augmentation or reduction, and no mammogram were excluded. Approximately 13% of breast cancer cases and 4% of controls were ineligible primarily because of death or pre-existent breast cancer. Of the 1,396 cases eligible to participate, 52.6% responded to the mailings and gave full consent. Of the 1,500 eligible controls, 48.7% responded to the mailings and gave full consent. After removing women who did not have suitable mammograms, the final sample consisted of 607 breast cancer cases and 667 control subjects. The original cohort and the nested case-control study were approved by the Committee on Human Studies at the University of Hawaii. All participants provided informed consent to participate in both studies.

### Data collection

Details of the study procedures were reported previously [4]. In brief, information on demographics, medical history, reproductive behavior, hormone replacement therapy (HRT) use, and body mass index (BMI) were collected with an extensive questionnaire at entry into the cohort during the period from 1993 to 1996 [13]. As part of the nested case-control study, a one-page Breast Health Questionnaire (BHQ) was completed to elicit information on menopausal status, previous breast surgery, mammography, and HRT medications [4].

### Mammograms

The mammographic films were retrieved from clinics located throughout the State of Hawaii using the authorization forms signed by the study participants. The original cohort study had no records on mammography use except for one item in the baseline questionnaire. At that time, 90% of Caucasian and Japanese women and 75% of Native Hawaiian women reported previous mammography [14]. Only craniocaudal views were digitized using a Kodak LS 85 Film Digitizer with a pixel size of 260 μm. If available, mammograms for every second or third year were scanned with the goal being to cover as wide a time period as possible for each woman. For cases, only mammograms taken before treatment for breast cancer were selected. However, the image of the contralateral breast taken at the time of diagnosis was used for five cases. The scanned images for both breasts were assessed for densities using Cumulus108 software [15] by one reader (GM), who was blinded to case status and time sequence of the mammograms. After the reader determined a threshold for the edge of the breast and for the edge of the dense tissue [15], the computer calculated the total number of pixels in the digitized image that constituted the total area and the dense area and computed the ratio between the two values as percentage density. Because readings for the right and left breast were very similar (correlation coefficient >0.90), we averaged the values for both to obtain one measure of total breast area, dense area and percentage density.

A random sample of 410 mammograms was read in duplicate to assess the reliability of the mammographic readings. The intraclass correlation coefficients [16] were 0.96 (95% confidence interval (CI) 0.95–0.97) for the size of dense area and 0.996 (95% CI 0.995–0.997) for the total breast area, resulting in an intraclass correlation coefficient for percentage density of 0.974 (95% CI 0.968–0.978).

### Statistical analysis

To test for differences in covariate values across breast cancer cases and control subjects, we performed *t* tests for continuous variables and χ^2 ^tests for categorical variables. We used unconditional polytomous logistic regression modeling with the SAS software [17] to compute odds ratios (ORs) and 95% CIs for the risks for DCIS and invasive cancer associated with breast density [18]. All *P* values reported are two sided. Breast cancer cases were divided into CIS and invasive based on information provided by the state-wide Hawaii Tumor Registry, a member of the National Cancer Institute's Surveillance, Epidemiology and End Results program. Of the 125 breast CIS cases, 119 were classified as having DCIS.

We chose two measures of mammographic breast density as our exposure variables: size of the dense area and percentage density. Percentage density was grouped into four commonly used categories: <10%, 10–24.9%, 25–49.9%, and ≥50%. The size of the dense areas was classified as follows: <15 cm^2^, 15–29.9 cm^2^, 30.0–44.9 cm^2^, and ≥45 cm^2^. Study participants were grouped into four categories: Japanese, Caucasians, native Hawaiians and others (mostly Filipinos). To maximize the number of participants per group, women of mixed ancestry – regardless of admixture – were assigned to one ethnic category according to the following priority ranking: native Hawaiian, Japanese, Caucasian, and, finally, other [13]. We created an HRT variable using the responses from the questionnaire at cohort entry and from the BHQ at enrollment into the breast density study. A comparison of the HRT information from the two questionnaires exhibited good agreement for overlapping years when both questionnaires reported HRT use. If a woman indicated that she had used HRT at any time but the write-in field in the BHQ was empty, we assigned the type of HRT from the cohort questionnaire completed at baseline. For the women with missing HRT type information (5.4%), we imputed the type based on hysterectomy status: estrogen only for women with a hysterectomy and combined therapy otherwise.

All models were adjusted for the following covariates that are known to be associated with breast cancer and mammographic density: mean age of all mammograms (continuous), ethnicity, BMI (<22.5, 22.5 to <25, 25 to <30, or ≥30 kg/m^2^), parity (0–1, 2–3, or ≥4), age at menarche (<13, 13–14, or ≥15 years), age at first live birth (<21, 21–30, >30 years, or no children), menopausal status (pre- or postmenopausal), family history of breast cancer (breast cancer in a first-degree relative or no history), and HRT use (never, estrogen only, or estrogen + progestin). Tests for trend were performed by fitting a variable representing ordinal categories (described above) of percentage density or dense area.

We were also interested in comparing how well percentage breast density and size of dense area predicted invasive breast cancer and breast DCIS. We performed unconditional binary logistic regression and examined the area under the receiver operating characteristic (ROC) curve, which is a method used in sensitivity-specificity analyses. The ROC curve assesses the ability of the model to distinguish between two groups (for example, diseased and disease free). If the model is able to separate the two groups perfectly, then the area under the ROC curve is equal to 1; if the model performs no better than chance, then the area will be 0.5.

## Results

Of all breast cancer cases, 119 (19.8%) were breast DCIS (Table 1). Japanese women had a greater proportion of breast DCIS than invasive breast cancer, and native Hawaiian women had almost twice the proportion of invasive cases compared with DCIS cases (*P *= 0.19). The mean age at diagnosis was similar for breast DCIS and invasive cases (*P *= 0.50). However, the breast DCIS cases had a greater proportion of postmenopausal women (89.9% versus 86.3% and 77.7% (for DCIS, invasive cases, and controls, respectively)) and had fewer children (2.2 versus 2.4 and 2.6 (for DCIS, invasive cases and controls, respectively)). Women with invasive breast cancer or breast DCIS had more mammograms than did controls (*P *< 0.0001). However, mean unadjusted age at first mammogram and last mammogram were not different across breast DCIS cases, invasive breast cancer cases, and controls (*P *= 0.32 and *P *= 0.66, respectively; results not shown). Although invasive breast cancer cases had a greater age-adjusted mean dense breast area than did breast DCIS cases (36.7 cm^2 ^and 34.9 cm^2^, respectively), the breast DCIS cases had higher percentage breast densities (38.2% and 36.5%, respectively) than did the invasive breast cancer cases because of their smaller mean total breast area.

Both percentage density and the size of the dense area were associated with breast DCIS and invasive breast cancer in our study (Table 2). For each cancer, trend tests were highly significant. Women with at least 50% percentage density had a 3.5-fold greater risk for invasive breast cancer than women with less than 10% density (OR 3.58, 95% CI 2.26–5.66) when compared with controls. The risk for DCIS was almost threefold greater in women with 50% or more percentage density than in women with less than 10% density (OR 2.86, 95% CI 1.38–5.94). For breast DCIS, the 95% CIs for the second and third categories of density included the null value. The comparison of breast DCIS with invasive breast cancer indicated a lower, although not statistically significant, risk for breast DCIS than for invasive cancer given the same level of percentage density. The risk estimates for the size of dense area in the breast were not as strong as they were for percentage breast density. For women with more than 45 cm^2 ^of dense breast area, the ORs of invasive breast cancer and breast DCIS were 2.92 (95% CI 2.01–4.25) and 2.86 (95% CI 1.57–5.20), respectively, compared with controls. The comparison of breast DCIS versus invasive breast cancer cases showed little difference.

Because of sample size limitations, we were only able to analyze mammographic density and risk for breast DCIS by ethnicity for Japanese and Caucasian women (results not shown). Both ethnic groups exhibited similar trends; risk for breast DCIS increased with increasing percentage density and dense area. However, the CIs were wide and, except for the highest category of percentage density and dense area in Caucasian women, all intervals included 1.

The area under the ROC curve was similar for percentage density and dense area. For the adjusted invasive breast cancer model, both values were 0.74, whereas for the adjusted breast DCIS model the areas under the ROC curve were 0.67. When modeled without adjustment for covariates, the area under the ROC curve for invasive breast cancer was 0.59 for both percentage density and dense area. A similar unadjusted model for DCIS yielded values of 0.59 for dense area and 0.61 for percentage density.

## Discussion

Our analyses revealed that mammographic breast density, as measured by percentage density and the size of dense area, is associated with breast DCIS as well as invasive breast cancer. The association was slightly weaker for breast DCIS than for invasive breast cancer. Comparisons of areas under the ROC curve indicated that breast density and dense area performed equally well in distinguishing breast DCIS and invasive breast cancer cases from controls. The ORs for breast DCIS in the present study were not as large as those reported in a study of a cohort of Canadian women [12], which estimated relative risks between 7 and 12 for detecting atypia/CIS in biopsy specimens from women with more than 50% density. However, the cell sizes within the strata of density were small in that study and cases were selected from a group of women with biopsies. A British case-control study [19] found much lower risks for breast CIS, and the estimates were similar in strength to those for invasive breast cancer. Women with Wolfe parenchymal patterns P2 or DY had 70% greater risk (95% CI 1.1–2.6) of screening detected *in situ *breast cancer compared with controls and a 30% greater risk (95% CI 1.2–1.8) of screening detected invasive breast cancer compared with controls.

Other studies of breast DCIS support the idea that there is a relation between *in situ *lesions and mammographic density. An investigation of 28 mammograms confirmed that breast DCIS occurs to a greater extent in areas of the breast that exhibit high mammographic density [11]. The relative risk for a second breast cancer was found to be three times higher among women with primary DCIS who had mammographic densities of 75% and greater compared with women with less than 25% density [20].

Although it is known that some breast DCIS lesions will develop into invasive breast cancers [21], and that DCIS is often found near invasive breast cancer lesions [22], it remains problematic to distinguish DCIS lesions that may progress to invasive breast cancer from those that may not. Given the growing number of breast CIS cases with increasing mammography screening [1], this question has important implications when decisions must be made regarding the aggressiveness of treatment [23]. A comparison of risk factors for CIS (ductal and/or lobular) and invasive breast cancer found mixed results. Established invasive breast cancer risk factors shown to be consistently associated with breast CIS include family history of breast cancer [24-30], low BMI among premenopausal women [24,29,30], and nulliparity [24,28,30,31]. However, other invasive breast cancer risk factors such as early age at menarche, late age at menopause, increased endogenous estrogen levels, and alcohol intake were associated with breast CIS in some studies [26,27,29,32], but not in others [24-27,30,33].

Our study and other reports mentioned above indicate that mammographic density is associated with breast CIS and with invasive breast cancer. However, because some studies describe differential effects for some risk factors, more research needs to be done. Studies with larger samples of breast CIS must be performed to assess whether factors such as postmenopausal obesity, growth factors (insulin-like growth factor-I), exogenous hormones, and cell proliferation biomarkers can help in elucidating the association between breast density, breast CIS, and progression to invasive breast cancer. New molecular techniques may have the ability to identify factors that are responsible for the progression from DCIS to invasive cancer [2].

A limitation of the present study is the lack of data on the frequency of mammography use. Although this population has high screening rates [14], we cannot rule out bias toward the null in estimates of DCIS risk because of the possibility of undetected breast DCIS among controls. We also had relatively low participation rates, although a comparison of participating and eligible women revealed that they had very similar characteristics [4]. Our assessments of HRT and BMI are limited because we had to rely on self-report, and assumed that their values remained constant between the time they were reported and when the study was completed, but an examination of BMI from a follow-up questionnaire five years after cohort entry showed that the mean BMI changes by only 0.50 kg/m^2 ^during that time. Therefore, differences in BMI are unlikely to change the results materially. The use of BMI in categories is unlikely to have confounded our results because analyses with continuous values of BMI gave nearly identical results. We had limited power to estimate the risk for DCIS; there were nearly four times as many invasive breast cancer cases as breast DCIS cases. Nevertheless, the cohort design, the multiple mammograms, and the frequent mammography use in this population must be considered strengths of this project. The high rate of mammography participation decreases the probability that a large number of cancers were missed in this population.

## Conclusion

In the present study similar patterns of association for mammographic density with DCIS and invasive breast cancers add to the growing body of evidence that certain breast CIS and invasive breast cancers may share etiologic factors. At the same time, it appears likely that factors that have not yet been identified may influence the effects of breast density and other known breast cancer risk factors in the progression of breast CIS to invasive breast cancer.

## Abbreviations

BHQ = Breast Health Questionnaire; BMI = body mass index; CI = confidence interval; CIS = carcinoma *in situ*; DCIS = ductal carcinoma *in situ*; HRT = hormone replacement therapy; OR = odds ratio; ROC = receiver operating characteristic.

## Competing interests

The authors declare that they have no competing interests.

## Authors' contributions

JG carried out the main statistical analysis, created variables used in this analysis, and drafted the manuscript. GM conceived of the nested case-control study, performed the mammographic density assessment, and helped with the draft and revisions of the manuscript. IP created a data set and variables used in analysis, assisted in writing of SAS programs, and advised on statistical analysis techniques. LK conceived of the Multiethnic Cohort and helped in revision of the manuscript. All authors read and approved the final manuscript.

## Figures and Tables

**Table 1:** Characteristics of the study population by disease status

Covariate	Invasive breast cancer	Breast DCIS	Controls	*P *value^a^
Sample size	482	119	667	-
Ethnicity (%)				0.19^b^
Hawaiian	14.3	8.4	24.3	
Japanese	46.5	55.5	43.8	
Caucasian	32.6	28.6	28.0	
Other	6.6	7.6	3.9	
Age at diagnosis (years)	63.5	62.9	N/A	0.50^b^
Age at recruitment (years)	60.0	59.6	56.7	0.58^b^
Mean age at all mammograms (years)	59.8	59.1	59.7	0.70
Body mass index (kg/m^2^)	25.2	24.5	25.7	0.07
Time from first mammogram to diagnosis (years)	6.2	6.7	N/A	0.17^b^
Family history of breast cancer (%)	16.8	16.8	12.0	0.05
Age at first birth (years)	25.0	25.1	24.7	0.65
Age at menarche (years)	13.1	12.9	13.1	0.47
Parous (%)	84.5	83.9	88.5	0.10
Number of children	2.4	2.2	2.6	0.02
Postmenopausal (%)	86.3	89.9	77.7	<0.0001
Ever HRT use (%)	67.2	73.9	69.3	0.01
Number of mammograms	3.2^c^	3.4^c^	2.4^c^	<0.0001
Breast percent density^d^	36.5	38.2	29.4	<0.0001
Breast dense area^d ^(cm^2^)	36.7	34.9	28.7	<0.0001
Total breast area^d ^(cm^2^)	114.5	106.9	118.9	0.08

Unless otherwise indicated, mean values are given. ^a^*P *ascertained from *t *test or χ^2 ^test, as appropriate. ^b^Compares only invasive breast cancer and breast DCIS cases. All other comparisons are of invasive breast cancer cases, breast DCIS cases, and controls. ^c^The range for number of mammograms is 11, 10, and 7 for invasive breast cancer, breast DCIS, and controls, respectively. ^d^Adjusted for age at recruitment. DCIS, ductal carcinoma *in situ*; HRT, hormone replacement therapy.

**Table 2:** Mammographic breast density and risk estimates for breast DCIS and invasive breast cancer

Exposure variable	Invasive breast cancer (*n*)	Breast DCIS (*n*)	Controls (*n*)	Invasive versus controls	DCIS versus controls	DCIS versus invasive
Mean percentage density (%)						
<10	63	18	158	1 (reference)	1 (reference)	1 (reference)
10–24.9	110	23	170	1.81 (1.21–2.70)	1.15 (0.57–2.30)	0.65 (0.31–1.35)
25–49.9	174	34	212	2.53 (1.69–3.78)	1.29 (0.64–2.59)	0.56 (0.27–1.17)
≥50	135	44	127	3.58 (2.26–5.66)	2.86 (1.38–5.94)	0.89 (0.41–1.91)
*P *value for trend				<0.0001	0.0010	
Mean breast dense area (cm^2^)						
<15	95	26	209	1 (reference)	1 (reference)	1 (reference)
15–29.9	136	29	200	1.58 (1.12–2.23)	1.05 (0.58–1.92)	0.75 (0.40–1.40)
30–44.9	109	28	133	1.93 (1.32–2.81)	1.70 (0.90–3.22)	0.92 (0.48–1.76)
≥45	142	36	125	2.92 (2.01–4.25)	2.59 (1.39–4.82)	0.99 (0.53–1.86)
*P *value for trend				<0.0001	0.0026	

Values are expressed as odds ratios (95% confidence intervals), which were estimated using polytomous logistic regression and adjusted for ethnicity, mean age of all mammograms, body mass index, age at first live birth, number of children, age at menarche, menopausal status, use of hormone replacement therapy, and family history of breast cancer. DCIS, ductal carcinoma *in situ*.
